# Body mass index for the assessment of adiposity in healthy aging Caucasian women: a dual-energy X-ray absorptiometry based study

**DOI:** 10.1007/s00296-026-06193-2

**Published:** 2026-07-22

**Authors:** Stavroula J. Theodorou, Daphne J. Theodorou, Ioannis Mitselos, Vassiliki Kigka, Andreas Fotopoulos, Andreas F. Mavrogenis

**Affiliations:** 1 Department of Radiology, University Hospital of Ioannina, Ioannina, Greece; 2 Department of Radiology, General Hospital of Ioannina, Ioannina, Greece; 3 Department of Internal Medicine, General Hospital of Ioannina, Ioannina, Greece; 4 Department of Nuclear Medicine, Bone Densitometry Section, University of Ioannina, Ioannina, Greece; 5 First Department of Orthopaedics, School of Medicine, National and Kapodistrian University of Athens, ATTIKON University Hospital, Athens, Greece

**Keywords:** Dual energy X ray absorptiometry, Obesity, Overweight, Body mass index, Fat body, Muscles, Body composition, Analysis, ROC

## Abstract

Accurate classification of obesity levels is of clinical importance for public health and decision making. Body mass index (BMI) is the most broadly used measure of obesity on a population scale. But, as with other diagnostic indicators, BMI can have potential pitfalls. We attempted to explore the diagnostic performance of BMI for assessing obesity. 330 healthy Caucasian women (native Greeks) (ages, 20 to 85 years) volunteers participated in the study. Dual-energy X-ray absorptiometry (DXA) was used to measure percentage of fat mass (%FM). The diagnostic performance of BMI was assessed using the widely used indicator for obesity of %FM > 35% in women. Receiver operating characteristic (ROC) analysis was performed to evaluate the ability of BMI to discriminate obesity from non obesity. The correlation between BMI and %FM, and lean mass (LM) and %LM, was also investigated. Logistic regression was performed to assess for associations between DXA-derived fat metrics and age. BMI-defined obesity (≥ 30 kg/m^2^) was present in 40.3% of women, while %FM DXA-defined obesity was present in 72% of the subjects. The largest discrepancy between BMI- and DXA-defined obesity was noted in women over 80 years (p = 0.0002). A BMI ≥ 30 kg/m^2^ had excellent sensitivity [99%, 95% confidence interval (CI), 95.8–99.9], but poor specificity [45%, 95% CI 38–52] to detect %FM-based obesity. BMI ≥ 25 kg/m^2^ had high sensitivity (92%, 95% CI 87.5–94.8) and good specificity (79%, 95% CI 68.7–86.6). The optimal cut point for BMI in the entire cohort found to correspond closer to the criterion value of 35%FM was 26.32 kg/m^2^ (p < 0.001). At this level, BMI had excellent sensitivity (96.7%, 95% CI 93.3–98.6) and moderate specificity (71.5%, 95% CI 62.4–79.5). In all women, BMI correlated better with %FM (r = 0.74 to 0.84, p < 0.05) than with %LM (r= −0.68 to −0.86, p < 0.05). Logistic regression analysis found age was a significant predictor of obesity (OR = 1.074, 95% CI 1.036–1.114, p < 0.05), increasing odds for excess adiposity by 7.4% each year. The diagnostic performance of BMI for assessing obesity is limited particularly in women with BMI ≥ 30 kg/m^2^. A BMI cut point ≥ 30 kg/m^2^ has very high sensitivity, but misclassifies as obese 55% of normal women. At the BMI cut off ≥ 25 kg/m^2^ sensitivity was high, but 21% of women were still misclassified as obese. A BMI cut point value of 26.32 kg/m^2^ has higher sensitivity and falsely classifies as obese 28% of the women. In light of these findings, revision of current BMI criteria for obesity screening may be warranted in white women of Mediterranean origin such as Greek women, and perhaps other populations.

## Introduction

The detrimental effects of osteoporosis on public health have universally been outperformed by overweight and obesity, an ongoing disease epidemic with a worldwide prevalence that exceeds 2.5 billion adults [1]. At the current accelerating rate of increase, by 2035, over half of the global population will be diagnosed with overweight (including obesity) [1]. In parallel with the global obesity crisis, there has been a rise in the risk for a plethora of co-morbidities, including type-2 diabetes, cardiovascular disease, metabolic syndrome, osteoarthritis, rheumatoid arthritis, and cancer [2]. Importantly, an incidental finding of overweight/obesity in a healthy young woman may have different clinical implications than the same diagnosis in an elderly woman with frailty, or a patient with “rheumatoid cachexia” reconciling the need using adipose tissue metrics for obesity screening [3, 4]. For example, fat mass measurements become particularly important for rheumatoid arthritis patients, who would receive targeted treatments given the reduced response rates for certain medications in obese individuals [5, 6]. Other than rheumatoid arthritis, however, common rheumatic disorders including psoriatic arthritis, systemic lupus erythematosus, and gout may be associated with sarcopenic obesity accompanied by disease progression and a poor functional performance [3, 7]. On the flip side of the coin, however, in overweight rheumatoid arthritis patients changes in body composition associated with chronic inflammation may as well relate to reasonable clinical outcomes, known as the “obesity paradox”. 

Current epidemiologic data are based on body mass index (BMI; weight in kilograms divided by the square of height in meters), an easy, non-invasive and low-cost anthropometric tool for defining obesity status. Collectively, several specific diagnostic BMI thresholds have been proposed for identifying or ruling out overweight and obese. In current clinical practice, overweight for individuals of European descent is defined at a BMI of 25 kg/m^2^ and obese at 30 kg/m^2^, with the latter generally corresponding to 35%FM in women [8–13]. Nevertheless, academic debate continues as whether %FM > 35% should be used as the gold standard to define obesity mainly due to limitations imposed by age-related variability in the distribution of fat [14–16]. Using BMI as a marker of obesity in the ELSA-Brasil cohort, researchers found excess weight had a universal and dose-response association with chronic musculoskeletal pain [17]. Because BMI confounds fat and muscle mass, however, and in addition provides no information about body fat distribution that is known to increase the risk of obesity-related morbidity, the clinical relevance ascribed to this body size indicator has been justifiably debated [2, 18]. For example, in patients with rheumatoid arthritis, increased visceral fat coupled with a normal BMI has been associated with high cardiovascular disease events, suggesting that BMI is an insufficient measure of clinical obesity [4]. Alternatively, direct fat mass (FM) measurement by dual energy X-ray absorptiometry (DXA) has been widely used to reduce risk for misclassification of obesity levels at the individual level and improve stratification of the individuals into groups according to the levels and severity of obesity [18]. In this regard, investigators have examined the performance of BMI with reference to DXA over a range of cut points (for BMI) to determine the clinical utility of BMI-based adiposity measures [10]. Regardless of the different BMI thresholds assessed, however, the need to define accurate, effective and clinically relevant BMI cut point levels based on %FM for classifying obesity levels is unmet. The purpose of this study is to explore the association between BMI and %FM, as measured on DXA scans in Caucasian women screened for overweight and obesity, and correlate BMI with whole-body composition measures.

## Materials and methods

### Study design and participants

Following approval from the Institutional Review Board with patient informed consent, a single-center prospective exploratory hypothesis-generating study was conducted in 330 healthy Caucasian women (of a Greek origin) who were recruited in a Mediterranean area [19]. Criteria for entry included age 20–85 years, Caucasian, good general health, normal ambulation, non pregnant, no metal implants, and ability to entirely fit with the DXA scanning field. Study participants were non-institutionalized, had no history of renal, hepatic or gastrointestinal disease, and were not using medications known to interfere with changes in body composition. Women with amenorrhea, malignancy, rheumatoid arthritis, current smoking, regular alcohol consumption, and vigorous physical training were excluded from study.

### Anthropometric measurements

Height (cm) and body weight (kg) of the participants were measured under fasting conditions. Participants wore only underwear and disposable paper shirts. Height (cm) was measured with a wall-mounted stadiometer and weight (kg) was measured with medical weighing scales (Seca 780 720, Hamburg, Germany). BMI was calculated as per WHO definition, in kg/m^2^ [8].

### Body composition measurements

Whole body DXA scan (Discovery W, Hologic, Waltham, MA) with software version V12.3.7 was performed to measure BMC (g), BMD (g/cm^2^), fat mass (g), and lean mass (g). The reported FM and LM values were calculated as the means. To eliminate differences between FM or LM as a result of different body mass among individuals, percentage FM or LM was also calculated. Percentage FM was calculated as FM divided by total mass (FM/FM + LM+BMC) x100, and percentage LM was calculated as LM divided by total mass (LM/FM + LM+BMC) multiplied by 100 for the total body. Total mass by DXA was highly correlated with body weight measured by medical scales (r = 0.999, p < 0.001). Measurements of each body composition parameter were made independently by an experienced investigator (with fellowship training in bone densitometry) who also calibrated the DXA using the spine phantom daily and the whole body phantom provided by the manufacturer at least once weekly to minimize the effects of operator influences. Precision error for total body FM and LM was 3.5% and 1.3%, respectively. All metal objects (jewelry, belts, etc.) were removed before the scan.

### Statistical evaluation

Linear regression analysis of body composition measures indicated sufficient conformity to normality. The data were stratified using ten-year intervals from age 20 to over 80 years. All measures were calculated as mean ± SD. Differences among age groups were tested for significance by two-tailed t test. Pearson correlation coefficient between BMI and %FM, BMI and LM and BMI and %LM was calculated and values were compared.

 The current %FM cutoff value of 35% or more was used to evaluate the diagnostic performance of BMI on obesity status [8, 12, 13]. For overweight, the cut point for BMI was 25–29.9 kg/m^2^ and for obesity the cut point BMI was ≥ 30 kg/m^2^ based on metrics taken from healthy reference population studies [12, 20]. Diagnostic performance of BMI to detect %FM-classified obesity was determined by calculating the following parameters: sensitivity, specificity, predictive values with their respective 95% confidence intervals (CIs) and likelihood ratios. Formulas for estimates were as follows: Sensitivity= True Positives (TP)/TP + False Negatives (FN). Specificity= True Negatives (TN)/TN + False Positives (FP). Positive Predictive Value (PPV) = TP/TP + FP. Negative Predictive Value (NPV) = TN/TN + FN. Plotting of a receiver operating characteristic (ROC) curve for BMI was performed both in the entire cohort and age-stratified groups, and the area under the curve (AUC) was calculated. Logistic regression analysis was used to evaluate the association of fat metrics with age in our study group. All analyses were conducted using MINITAB for Windows (Version 15, State College, PA) and SPSS for Windows (V26; SPSS Inc, Chicago, IL). The 0.05 level of significance was used for all statistical tests.

 For internal validation, we performed bootstrap resampling analysis (1000/5000 replications) yielding a robust estimate with low standard error (SE = 0.004) and high precision. Bootstrap bias was minimal at 0.612. Resampling metrics confirmed that the model parameters were stable and not influenced by outliers or overfitting.

## Results

Table 1 presents the baseline anthropometric and densitometric characteristics for each age group. The mean age ± standard deviation (SD) of all 330 women was 52.7 ± 18.6 years. Measures showed that with advancing age there was progressive decrease in height of these women across all ages, with most significant changes after age 40 (p < 0.001). The mean BMI ± SD was 28.8 ± 5.8 kg/m^2^, and the mean %FM ± SD was 38.2 ± 6.7. Based on BMI classification (≥ 30 kg/m^2^) of obesity, the prevalence of the condition in our study participants was 40.3%. When using FM > 35% for obesity, 72% of these women were identified as obese. DXA-derived skeletal muscle measures showed significant decrease in lean mass after age 60 (p < 0.01).

**Table 1:** Anthropometric and DXA-based measurements (mean ± SD) by decades of age

Age in decades	*N*	Weight kg	Height cm	BMI Kg/m^2^	%FM	LM kg	%LM
20–30	51	62.9 ± 14	163.7 ± 5.8	23.4 ± 5^*^	31.9 ± 7.8^*^	39.6 ± 5.5	64.5 ± 7.4^*^
31–40	45	67.8 ± 15.5^*^	162.7 ± 6^***^	25.5 ± 5.4^***^	35.8 ± 6.5^*^	40.4 ± 6.1	61 ± 6^*^
41–50	54	74.4 ± 15	158.1 ± 6	29.6 ± 5.1	38.7 ± 5.8	42.6 ± 6.9	58.3 ± 5.5
51–60	55	75.7 ± 15.5	156.8 ± 5.9^**^	30.7 ± 5.5	39.8 ± 5.5	42.6 ± 6.3	57.4 ± 5.7
61–70	50	76.6 ± 12.5^**^	153.2 ± 4.6	32.7 ± 5.4^*^	41.5 ± 4.3	42.4 ± 5^**^	55.9 ± 4.1
71–80	50	70.3 ± 11.5^**^	151.8 ± 6.8^*^	30.4 ± 4.2	41 ± 5.8	39.1 ± 5^*^	56.4 ± 5.6
81–85	25	63.5 ± 8.9	148.9 ± 5.7	28.7 ± 3.8	38.7 ± 5.9	36.9 ± 3.2	58.9 ± 5.7

*N* number of participants, *BMI* body mass index, *FM* fat mass, *LM* lean mass

*p < 0.05; **p < 0.01; ***p < 0.001 as compared to subsequent age group

### Diagnostic performance of BMI

Table 2 shows the parameters of the diagnostic performance of BMI for obesity using *three* cut points at ≥ 25 kg/m^2^, *26.32 kg/m*^*2*^, and at the cut point ≥ 30 kg/m^2^. A BMI cut point of ≥ 25 kg/m^2^ had high sensitivity (true-positive rate) (92%, 95% CI 87.5–94.8) and good specificity (79%, 95% CI 68.7–86.6) to detect %FM-defined obesity. A BMI ≥ 30 kg/m^2^ had an excellent sensitivity [99%, 95% CI 95.8–99.9] and a poor specificity [45%, 95% CI 38–52] to detect %FM-defined obesity.

**Table 2:** Measures of diagnostic performance of BMI for detecting obesity at the cut points of ≥ 25 kg/m^2^, 26.32 kg/m^2^, and ≥ 30 kg/m^2^

Diagnostic performance metrics	BMI ≥ 25 kg/m^2^	BMI 26.32 kg/m^2^	BMI ≥ 30 kg/m^2^
Sensitivity (%)	92 (87.5–94.8)	96.7 (93.3–98.6)	99 (95.8–99.9)
Specificity (%)	79 (68.7–86.6)	71.5 (62.4–79.5)	45 (38–52)
Adiposity prevalence	73 (67.9–77.7)	64.8 (59.4–70)	40.3 (34.9–45.8)
PPV (%)	92 (88.6–94)	86.2 (82.4–89.3)	55 (52–58)
NPV (%)	78 (69–84)	92.2 (85–96.1)	99 (92–99.8)
+LR	4.3 (3–6)	3.4 (2.5–4.5)	1.8 (1.6–2)
−LR	0.1 (0.07–0.16)	0.05 (0.02–0.1)	0.02 (0–0.1)

The 95% CIs are shown in parentheses

*BMI* body mass index, *PPV* positive predictive value, *NPV* negative predictive value, +LR positive likelihood ratio, −LR negative likelihood ratio

**Fig. 1:**
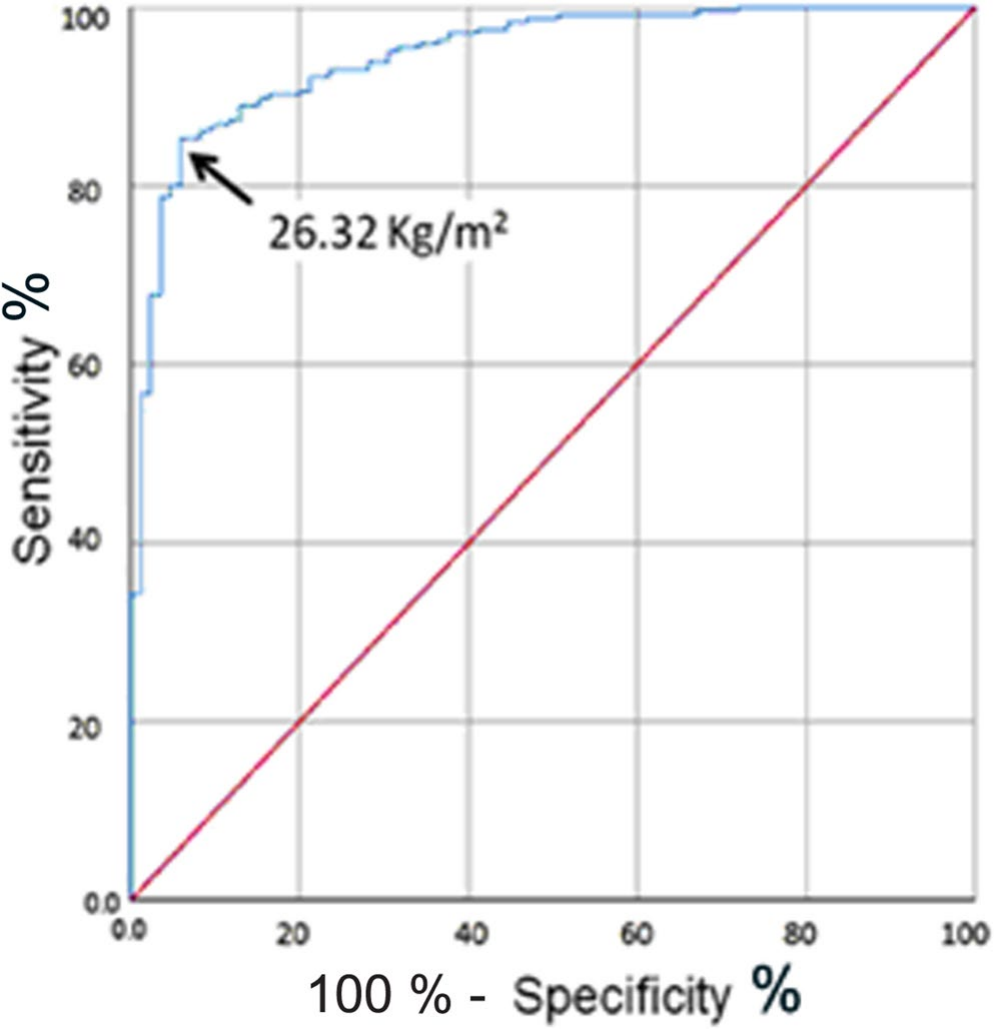
ROC curve for BMI versus %FM-defined obesity. BMI 26.32 kg/m^2^ is the cut point that corresponds to the best tradeoff between sensitivity and 1-specificity

For the entire cohort, the ROC curve for BMI to detect an excess in %FM (> 35%) and the diagnostic performance for the best identified BMI cut point is shown in Fig. 1.

We found that the AUC was 0.947 (p < 0.001) for BMI to detect %FM > 35%. The best cut point for BMI was 26.32 kg/m^2^, indicating a combination of excellent sensitivity (96.7%) and moderate specificity (71.5%). The AUC 95% CI was 0.92 to 0.97 suggesting that even at the lower limit of 0.92 the diagnostic ability of the BMI cutoff 26.32 kg/m^2^ is good. Table 3 is the contingency table of test results used to calculate sensitivity and specificity for BMI 26.32 kg/m^2^ cut off.

**Table 3:** Contingency table comparing the BMI cut off values of 26.32 kg/m^2^ against the 35% fat mass (FM) criterion

	FM ≥ 35%	FM < 35%	Total
BMI > 26.32 kg/m^2^	207 (TP)	7 (FN)	214
BMI < 26.32 kg/m^2^	33 (FP)	83 (TN)	116
Total	240	90	330

*BMI* body mass index, *FM* fat mass, *TP* true positive, *FP* false positive, *FN* false negative, *TN* true negative

For the entire cohort of women, the ROC curve for BMI to detect an excess in %FM (> 35%) and the diagnostic performance for the best identified BMI cut point is shown in Fig. 1. We found that the AUC was 0.947 (p < 0.001) for BMI to detect %FM > 35%. The best cut point for BMI was 26.32 kg/m^2^, indicating a combination of excellent sensitivity (96.7%) and moderate specificity (71.5%). The AUC 95% CI was 0.92 to 0.97 suggesting that even at the lower limit of 0.92 the diagnostic ability of the BMI cutoff 26.32 kg/m^2^ is good. Particular age-stratified ROC analyses showed that in Greek women 20–30 years, the BMI cut off was 23.97 kg/m^2^. For the age group 31–40 years, the BMI cut off was 25.08 kg/m^2^. In middle-aged women 41–50 years the obesity BMI threshold was 26.38 kg/m^2^. For women aged 51–60 years the BMI cut off was 25.88 kg/m^2^. Women aged 61–70 years had a BMI threshold 27 kg/m^2^. For the age bracket 71–80 years the BMI threshold was 27.35 kg/m^2^. Old women aged 81–85 years had a BMI cut off value 26.80 kg/m^2^.

Changes in adiposity prevalence at different BMI cut offs consistently linked to predictive values, in the studied female population. Using logistic regression, age was a significant predictor of obesity based on the DXA-derived fat tissue metrics. For our women cohort, the odds ratio (OR) for excess adiposity was 1.074 [95% CI 1.036–1.114, p < 0.05], suggesting that each year of increasing age is associated with an increased 7.4% risk of obesity.

### Discrepancy analyses by decade of age

The discrepancies (15% to 44%) between BMI- and DXA-determined obesity by age-group are presented in Table 4. Elderly women (over 80 years) had the largest discrepancy (44%) between BMI and %FM, while young women (in their 20 s) had the smallest discrepancy (15%) between measurements of obesity obtained with these two methods. There was significant discrepancy (*p* = 0.00001–0.004) between BMI- and DXA-determined obesity for all age groups that resulted in different diagnostic ranking of the severity of obesity. Discrepancy was largest in older women over 80 years of age (*p* = 0.0002).

**Table 4:** Percent discrepancy (%D) between BMI- and DXA- based obesity status by age

Age group	BMI obese number (%)	%FM obese Number (%)	%D
20–30	8 (2.4%)	16 (4.8%)	15**
31–40	9 (2.7%)	27 (8%)	40***
41–50	25 (7.5%)	40 (12%)	28**
51–60	25 (7.5%)	46 (14%)	38***
61–70	29 (8.7%)	46 (14%)	34***
71–80	28 (8.4%)	45 (13.6%)	34***
81–85	9 (2.7%)	20 (6%)	44***

BMI and DXA measures cell inputs are raw numbers of women. % D (discrepancy) is the percentage of obese women defined by BMI-obese women defined by DXA

** *p* < 0.01, *** *p* < 0.001

### Correlation between BMI and %FM, LM, and %LM

The correlations between BMI and %FM vs. BMI and both LM and %LM by age group are shown in Table 5. Overall, BMI had a strong correlation with %FM (r = 0.81, p < 0.001) and a moderate correlation with LM (r = 0.65, p < 0.001). In particular, we found that BMI correlated better with %FM than with LM across all age groups except during 41–50 and 61–70 age brackets. Variability assessment of %FM for a given BMI value (i.e., 25 kg/m^2^) showed that the distribution of %FM ranged widely from 24.8% to 43.2%. We performed sub-analyses to evaluate the correlation between BMI and %FM, BMI and LM and BMI and %LM in the overweight (BMI, 25–29.9 kg/m^2^) women. Although correlation of BMI with %FM was weak (r = 0.34, p < 0.001) BMI still had a better correlation to %FM than to LM (r = 0.21, p = 0.028). The correlation between BMI and %LM in overweight women became weak (r= −0.31, p = 0.001). For the obese subgroup (BMI ≥ 30 kg/m^2^), there was moderate correlation of BMI with %FM (r = 0.44, p < 0.001) and LM (r = 0.47, p < 0.001). There was moderate correlation between BMI and %LM (r = −0.46, p < 0.001) for obese women. In comparing the BMI to %LM in the whole group, there was a strong correlation (r = −0.80, p < 0.001).

**Table 5:** Correlation coefficients between BMI and %FM, LM and %LM by age in decades

Age in decades	BMI-%FM	BMI-LM	BMI-%LM
20–30	0.848	0.730	−0.838
31–40	0.844	0.786	−0.829
41–50	0.709	0.775	−0.688
51–60	0.744	0.723	−0.746
61–70	0.741	0.829	−0.714
71–80	0.752	0.484	−0.734
81–85	0.874	0.435	−0.867

All correlations significant at *p* < 0.05

## Discussion

Overweight and obesity are increasingly common in young individuals and adults, and the resulting impact on adverse health outcomes is immense [1, 11, 18]. Recently, the need for more precise approach to obesity was met by the new definition and diagnostic perspective that distinguishes “clinical obesity”, an established disease state, from “preclinical obesity”, carrying an increased future health risk [18, 21]. Foremost, implementation of this new diagnostic framework into patient care can be used to monitor disease progression on individuals living with obesity and as an additional prognostic variable. For example, effective integration of fat metrics to help screen for obesity in rheumatology patients is particularly important for those individuals who would undergo treatment with biological and targeted synthetic disease-modifying drugs [5, 6]. Indeed, recognition of excess adiposity is crucial in rheumatology patients because obesity can be associated with poor outcomes [3, 5, 6]. In one study of 361,952 participants from the UK Biobank cohort, 1 SD increase in BMI was found to cause an increased risk for developing rheumatic disease including rheumatoid arthritis, osteoarthritis, psoriatic arthropathy, gout, and inflammatory spondylitis [22]. In another study of 183 individuals with clinical symptoms of fibromyalgia, tender points showed significant positive correlation with higher BMI levels [23]. Outside of the scope of this investigation, adiposity measurements have also been used to screen for overweight in older adults with various disease states including osteoporosis, sarcopenia, several cancers, and cardiometabolic diseases such as type 2 diabetes, coronary artery disease and cerebrovascular disease, providing further benefit to patients at risk for obesity-related morbidity or mortality [18, 24, 25]. Before stratification of clinical obesity can be widely adapted to medical practice however, further agreement on what measurements for identification of obesity are most clinically relevant is warranted.

 Although BMI is the single most common anthropometric measurement for body size used in epidemiologic studies, there are important caveats: these are not measurements of genuine adiposity. For example with ageing, there is a general increase in adiposity and decline in lean mass (sarcopenia) and thus, older people may present with excess fat deposits, despite ranging within the normal or overweight BMI. Clinical complexity of obesity imposes adjustments of BMI measurements for age, sex, and genetic and ethnic backgrounds [24, 26]. In this study, DXA-based measurements showed that age was a significant predictor of obesity, with each additional year increasing odds of excess adiposity by 7.4%. Whole-body lean mass (LM) decreased significantly (sarcopenia) after 60 years of age in healthy BMI-defined overweight or obese women. We found body height loss (reflecting bone loss) across all ages in normal, overweight, or obese women accentuated after age 40 years. These findings, however, are not unique compared to previously discussed studies of obesity risk rising with age [27, 28]. Collectively, our results representing the variable combinations of sarcopenia, osteoporosis, and adiposity are thought to relate to the interaction between muscle, bone, and fat that can be affected by aging, genetics, and lifestyle [24, 29]. Another important caveat: the use of BMI as an index of adiposity lacks information about fat tissue distribution in the body that may have meaningful clinical implications. Given these major limitations of BMI-based measurements, other simple measurements of body size have been used including waist circumference, waist-to-hip ratio, and waist-to-height ratio [30]. As an alternate, the implementation of DXA for medical assessment of obesity has allowed for direct measures of body fatness, which are fairly standardized [31]. In a previous study of female patients with rheumatoid arthritis, investigators found the frequency of clinical obesity as determined by BMI was 33.7%, 89.9% by waist circumference, and 56.1% by DXA (the gold standard for measuring body fat), suggesting that BMI may ultimately not be accurate for the measurement of adiposity [32].

 In comparing BMI and DXA metrics of adiposity in our study, we found that BMI has limited diagnostic relevance to identification of subjects with increased body fatness, particularly those with BMI ≥ 30 kg/m^2^. Although there was excellent sensitivity, NPV and negative likelihood ratio of BMI ≥ 30 kg/m^2^ for identifying obese women, BMI had a very poor specificity, massively overclassifying more than half (55%) of women without %FM-defined obesity. These results are in line with prior work that has shown BMI is not sufficiently accurate in diagnosing increased body fatness in obese subjects [12, 33]. In contrast, other studies have found that a BMI cut point ≥ 30 kg/m^2^ has low sensitivity and good specificity, misclassifying almost half of %FM-determined obese women [12]. In theory, BMI has an overall very good correlation with %FM (r = 0.79) [34]. In our study, BMI had strong correlation with %FM and also correlated moderately with LM. BMI correlated better with %FM than with LM, across all age groups except for the 41–50 and 61–70 age brackets. Our findings support earlier work suggesting that for any given BMI value there is high inter-subject variability in %FM, likely reflecting different instrumentation, and ethnic and environmental discrepancies [35, 36]. When LM was adjusted for body mass (%LM), we found that the discrepancies for these age groups were attenuated and BMI correlated better with %FM (p < 0.05). It is likely that use of %LM eliminates the effect of different body size among individuals which impacts the correlation of BMI with FM and LM.

 Regardless of the wide use of BMI as an index of adiposity, academic debate continues about the proposed different thresholds used for diagnosing obesity. Largely, the use of BMI in defining obesity is arguable owing to unclear contributing causes and their individual influence on the deposition of body fat. Some researchers have found that BMI is an acceptable surrogate measure of body fatness for population studies, but it is inferior to direct measures of FM at an individual level [37]. Other researchers have found that in white women a BMI of 23 kg/m^2^ might provide a better diagnostic screening cut point for obesity than the WHO-based definition [38]. In particular, for young women 20–45 years old, BMI has proved a sensitive measure for diagnosing obesity based on a 33% FM criterion value [38]. In our study with a 35% FM criterion, we found that BMI cutoff at 26.32 kg/m^2^ provided the optimal tradeoff between high sensitivity (96.7%) and moderate specificity (71.5%), allowing for reasonable screening of obesity in women. Other authors have reported that 25.5 kg/m^2^ is the optimal cut point in women over 50-years old, and 27.3 kg/m^2^ in women aged 16 to 80 years [39, 40]. In contrast, Hortobagyi et al. using a cut point of BMI > 27 kg/m^2^, found 27% sensitivity and 98% specificity in hydrodensitometry-derived FM measurements [41]. Similarly, in a study of Saudi women, Alammar et al. suggested that the best BMI cut point should be lowered to 27 kg/m^2^ [20]. We observed that the BMI cut point ≥ 30 kg/m^2^ would falsely classify 55% of women as obese. As a result, normal women would have received unnecessary treatment for body weight control, with negative ramifications on health-care systems and societal costs [18]. Moreover, for BMI ≥ 25 kg/m^2^ in our study population, 21% of individuals were misclassified as (falsely) obese. In this sense, the wide range of BMI thresholds reflects the need for developing an accurate and effective tool that classifies obesity levels in the clinical health level [2, 11, 21].

 Age-stratified ROC analysis showed that the existing BMI cut off of ≥ 30 kg/m^2^ for obesity often proves inadequate. For example, in young Greek women 20–40 years of age, the optimal BMI cut off is lower (23.97–25.08 kg/m^2^) falling in the upper normal weight and the lower overweight range on the basis of standard BMI classification. Similarly, for middle-aged Greek women (41–60 years) the obesity BMI threshold is still lower (25.88–26.38 kg/m^2^), falling again in the overweight category as compared to the traditional BMI cut off. In older women (61–80 years), ROC analyses suggested lowering the obesity BMI threshold to approximately 27 kg/m^2^ for diagnosing obesity. Finally, in the extreme old age (81–85 years) further lowering of the obesity BMI threshold to 26.8 kg/m^2^ is warranted. These results underscore that the capability of BMI 30 kg/m^2^ to detect obesity in Greek women varies with age, requiring adjustments with lowering of existing thresholds. Ultimately, age-specific BMI cut-offs may improve performance of that somatometric index for oprtimized detection of obesity as opposed to a universal BMI cutoff value.

 Comparing measurements between different techniques provides some context. Our study supports the dramatic increase in prevalence of obesity between BMI-defined obesity calculated in 40.3% of women as opposed to 72% when %FM > 35% was employed. Yet use of the best identified cut point for obesity (BMI ≥ 26.32 kg/m^2^) in our women results in misclassification of 28% of the women as obese. But again, using the WHO threshold for BMI ≥ 30 kg/m^2^ we misclassify (17/330) 5% of women with excess adiposity as being normal, missing the opportunity to properly address health risks in these individuals [18]. In practice, two individuals with identical BMIs may have different %FM, and these differences may be accentuated if they vary in ethnicity, age and gender [26]. This misclassification of obese and non-obese women may relate to the intrinsic inability of BMI to discriminate between FM and LM, taking into account also that LM possesses the numerator site in the BMI fraction that can strongly impact results [26]. It is also important to consider that the diagnostic discordance between BMI- and DXA-defined obesity is influenced by age as evidenced mostly in women older than 80 years. Similar results were also reported by Romero et al. [12] who found that the diagnostic performance of BMI diminishes with age, likely owing to an increase of FM relative to LM in aging women. In this regard, we found that age is a critical predictor of obesity evolving across the lifespan of women. Prior work by our group and others also did show increasing adipose depots with advancing age [14–16, 19, 42]. Although not appropriate for assessing age-related differences in body fat distribution (as opposed to DXA), however, BMI remains a universally accepted measure of adiposity, with clinical relevance [18, 27, 43].

 The currently used cut point for obesity (BMI ≥ 30 kg/m^2^) is based on observational studies in Europe and the United States that have assessed the relationships of morbidity and mortality with BMI [9]. In this respect our findings are in accordance with those in other studies indicating that a universal BMI cut point is not an appropriate criterion for diagnosing obesity, because its use can lead to substantial errors [2, 44, 45]. To gain greater reliability, definitions of BMI cut points for obesity would likely be needed to be population-specific [46, 47]. Indeed, there are different BMI cut point values set within different western populations. For example, in a study of Spanish women the BMI cut point for obesity was 27.4 kg/m^2^ associated with 35%FM [48], while in both American Caucasian and European Caucasian women the threshold raised to 30 kg/m^2^ [49]. We observed a lower BMI defining cut point for women at 26.32 kg/m^2^ corresponding to %FM > 35%. Also, for Asian people, WHO has suggested lowering the BMI cut off for diagnosing obesity to 27 kg/m^2^ due to higher %FM compared to Caucasians [50]. Previously documented influences of numerous factors including energy balance and variable body built among different ethnic groups are likely to relate to differences in the %FM and BMI relationship [36].

 Although there are drawbacks in BMI-based diagnosis of obesity mostly related to the fact that BMI does not merely represent FM, BMI is the simplest metric for evaluating fatness also operating as a screening tool. Usually, increments in body weight or BMI represent fat gain, with the exception of muscular athletes, subjects with heart or renal failure and ascites, having increased volume of third space that may cause spurious increases in BMI that in turn, result in overdiagnosis of obesity. Conversely, spurious decreases in BMI and underdiagnosis of obesity may be present in individuals who have lost bone or muscle mass, elderly women or people of certain ethnicities [18]. We found that the cut point for BMI ≥ 25 kg/m^2^ which we observed in our study has both high sensitivity and PPV for diagnosing obesity. Better yet, the proposed BMI of 26.32 kg/m^2^ is the best cut point in our study population for identifying obese women.

 Our study has some potential limitations. First, we used as reference the gold standard definition of obesity by WHO (BMI≥ 30 kg/m^2^) that is less than accurate for determining the biological continuum of fat tissue deposition. We attempted to define a plausible threshold for the diagnosis of obesity in light of this inherent limitation. Second, we analyzed measurements only in women. Concerns about variability in measurements of BMI between men and women may restrain generalizability of our results. Also our study includes only Caucasian adult subjects of a Greek origin and as such our results may not allow extrapolation to other populations. Variability has been shown between %FM and BMI among different ethnic groups [44, 45]. Clinical parameters including menopause or other variables influencing body composition (i.e., physical activity) other than anthropometric measures are not defined in our cohort. Changes in fat mass are associated with age rather than menopause [27]. We feel, however, that the study group is fairly representative of healthy community-dwelling women assessed by whole-body DXA examination, at a major, tertiary care university medical center. Future studies within the same ethnic group, or larger, independent cohorts are needed to further explore age-stratified analyses and validate the proposed thresholds, confirming robustness of our findings. Lastly, sample size calculation was not performed in our study. We elected to perform internal validation via bootstrapping instead, yielding highly stable parameter estimates with a markedly low standard error. This study also has several strengths. First, our population is homogeneous in terms of gender and ethnicity of the participants, eliminating potential inequities introduced by these factors to increase the internal validity of the study (yet reducing generalizability of the results). Second, the characteristics of our study population are representative as we included ages from early adulthood to very old age and an extended range of BMIs. Finally, current %FM measures were obtained using DXA, a widely used reference standard method that has been validated for body composition analysis (including fat tissue measurements).

 At this time, recognition of obesity as a major disease state is still in its infancy, which undermines clinical decision making and public health strategies. In light of the need for redefining obesity our study documents the extent to which BMI as a universal and practical indicator of obesity needs to be updated. For BMI, we analyzed the performance of three cut points: the widely accepted cut point of ≥ 30 kg/m^2^, the cut point ≥ 25 kg/m^2^, and a proposed cut point of 26.32 kg/m^2^ fitting best the ethnic group of Greek women for obesity screening purposes. These findings highlight the need to interpret standard BMI thresholds cautiously. In this context, revision of current BMI criteria in white Mediterranean women of a Caucasian origin, or more diverse cohorts may be justified in better defining obesity status. Future studies should address optimal BMI thresholds in different patient populations for accurate disease classification of obesity at the individual and the public health context.

## Data Availability

Data for this manuscript are not openly shared.
